# Standalone Smartphone Cognitive Behavioral Therapy–Based Ecological Momentary Interventions to Increase Mental Health: Narrative Review

**DOI:** 10.2196/19836

**Published:** 2020-11-12

**Authors:** Marta Anna Marciniak, Lilly Shanahan, Judith Rohde, Ava Schulz, Carolin Wackerhagen, Dorota Kobylińska, Oliver Tuescher, Harald Binder, Henrik Walter, Raffael Kalisch, Birgit Kleim

**Affiliations:** 1 University of Zurich Psychiatric University Hospital Zurich Switzerland; 2 Jacobs Centre of Productive Youth Development University of Zurich Zurich Switzerland; 3 Charite Berlin Universitätsmedizin Berlin Germany; 4 University of Warsaw Warsaw Poland; 5 Gutenberg University Medical Center Mainz Germany; 6 Institute for Medical Biometry and Statistics Faculty of Medicine University of Freiburg Freiburg Germany

**Keywords:** mHealth, mobile app, ecological momentary intervention, EMI, cognitive behavioral therapy, CBT, COVID-19, mobile phone, smartphone

## Abstract

**Background:**

A growing number of psychological interventions are delivered via smartphones with the aim of increasing the efficacy and effectiveness of these treatments and providing scalable access to interventions for improving mental health. Most of the scientifically tested apps are based on cognitive behavioral therapy (CBT) principles, which are considered the gold standard for the treatment of most mental health problems.

**Objective:**

This review investigates standalone smartphone-based ecological momentary interventions (EMIs) built on principles derived from CBT that aim to improve mental health.

**Methods:**

We searched the MEDLINE, PsycINFO, EMBASE, and PubMed databases for peer-reviewed studies published between January 1, 2007, and January 15, 2020. We included studies focusing on standalone app-based approaches to improve mental health and their feasibility, efficacy, or effectiveness. Both within- and between-group designs and studies with both healthy and clinical samples were included. Blended interventions, for example, app-based treatments in combination with psychotherapy, were not included. Selected studies were evaluated in terms of their design, that is, choice of the control condition, sample characteristics, EMI content, EMI delivery characteristics, feasibility, efficacy, and effectiveness. The latter was defined in terms of improvement in the primary outcomes used in the studies.

**Results:**

A total of 26 studies were selected. The results show that EMIs based on CBT principles can be successfully delivered, significantly increase well-being among users, and reduce mental health symptoms. Standalone EMIs were rated as helpful (mean 70.8%, SD 15.3; n=4 studies) and satisfying for users (mean 72.6%, SD 17.2; n=7 studies).

**Conclusions:**

Study quality was heterogeneous, and feasibility was often not reported in the reviewed studies, thus limiting the conclusions that can be drawn from the existing data. Together, the studies show that EMIs may help increase mental health and thus support individuals in their daily lives. Such EMIs provide readily available, scalable, and evidence-based mental health support. These characteristics appear crucial in the context of a global crisis such as the COVID-19 pandemic but may also help reduce personal and economic costs of mental health impairment beyond this situation or in the context of potential future pandemics.

## Introduction

### Prevalence of Psychiatric Disorders

Heightened prevalence of psychiatric disorders is one of the largest challenges for modern health care. In 2001, the World Health Organization (WHO) estimated that 1 in every 4 individuals worldwide was affected by one or more mental or neurological health problems during their lives [1]. Prospective longitudinal studies suggest that the vast majority of people will have a mental health disorder at some point in their lives [2,3]. In 2017, >970 million people were diagnosed with mental or substance use disorders [4]. These numbers likely represent a gross underestimate because of underreporting for fear of stigma or limited mental health literacy, limited access to therapy services, high costs of treatment, and additional reasons, such as recent conditions caused by the COVID-19 outbreak.

The global COVID-19 pandemic is likely to further increase the need for mental health care interventions and new ways of implementing them. Global pandemics cause high levels of stress and may lead to mental health problems, such as depression, anger, anxiety disorders, and posttraumatic stress disorder, as well as an increase in smoking and alcohol consumption [5,6]. Current measures of physical distancing and quarantine aimed at curbing the spread of COVID-19 likely have additional detrimental psychological side effects, including loneliness [7]. At the same time, people are considerably less likely to receive professional face-to-face psychological help to overcome their fears, lowered mood, and other mental health problems [8].

Given the low detection rates of mental health problems, the WHO estimates that 76% to 85% of people in low- and middle-income countries and 35% to 50% of people in high-income countries receive no treatment for their mental health disorder [9]. After the pandemic is over, these numbers will be even higher. Moreover, those who seek treatment often do so only years into the mental disease, meaning that comorbid disorders and difficulties in many domains of life, including family and work, have already developed [10].

### Ecological Momentary Interventions

The rapid growth in the use of smartphones has created a new branch of medicine—mobile health (mHealth). Currently, the most popular solutions in mHealth are mobile apps because they are easy to use and are widely available [11]. Such apps typically use ecological momentary intervention (EMI) to deliver treatments provided to people in their everyday lives [12]. This approach captures and modifies specific moment-to-moment situations that emerge in the real world rather than targeting problematic thoughts, emotions, and behaviors within therapy sessions or in the hospital [13,14].

### Cognitive Behavioral Therapy

Most of the scientifically tested apps are based on cognitive behavioral therapy (CBT) principles. CBT was first created and established by Beck [15] and Ellis [16] and is based on the theory that maladaptive cognitions, such as general beliefs and automatic thoughts about the self and the world, contribute to the maintenance of emotional distress and behavioral problems. Accordingly, CBT specifically targets these maladaptive cognitions and behaviors. CBT is one of the most extensively used and researched form of psychotherapy [17] and is considered the gold standard for the treatment of many mental health problems [18]. Owing to their strong empirical foundation and clear structure, CBT-based interventions are well suited for application in mHealth. Here, we examine mobile apps that have been designed using a *rational app design*. With this definition, we refer to apps that are based on a CBT rationale and implement established and empirically validated CBT tools and techniques.

### Objectives

Several reviews and meta-analyses reported the efficacy of EMIs for several mental health problems, including depression, anxiety, perceived stress, and eating disorders [12,19-23], but they were not focused solely on CBT-based interventions. Other reviews have focused on or have not excluded blended treatments such as face-to-face psychotherapy in combination with an app [24,25]. However, to the best of our knowledge, there are currently no reviews on the efficacy, effectiveness, and feasibility of standalone EMIs delivered via mobile apps and following a rational app design, in both healthy and clinical populations. However, the rapid development of this type of psychological support and recent pandemic-quarantine circumstances have created an urgent need for a comprehensive summary of the studies published so far.

## Methods

### Search Strategy

To build a comprehensive overview of the existing literature, 4 search terms were used to identify articles: (1) mental health, (2) smartphones, (3) CBT, and (4) ecological momentary interventions (see Multimedia Appendix 1 for the complete search strings) in the MEDLINE, PsycINFO, EMBASE, and PubMed databases on January 16, 2020.

### Inclusion Criteria

The following criteria were used to select the studies: (1) peer-reviewed publications; (2) written in English; (3) published between January 1, 2007, and January 15, 2020; (4) standalone treatments (blended interventions were excluded); (5) explicitly aiming to increase mental health; and (6) focusing on feasibility, efficacy, and/or effectiveness of EMI. Both within- and between-group designs and studies with both healthy and clinical samples were included.

The time range was decided based on the launch date of the first App Store in 2007. This means that the review proposes an overview of presumably all the papers published since the development of the field started.

### Quality Assessment

For selected studies, we assessed the methodological risk of bias of included studies in accordance with the Cochrane Handbook [26] and the guidelines of the Cochrane Consumers and Communication Review Group [27], which recommends the explicit reporting of the following individual elements for the studies: random sequence generation, allocation sequence concealment, blinding (participants and personnel), blinding (outcome assessment), completeness of outcome data, selective outcome reporting, and other sources of bias. Overall, from the 14 randomized controlled trials (RCTs) included, 8 were rated as low risk and 6 as medium risk. From the remaining 12 non-RCT studies, 11 were rated as medium risk and 1 as high risk. The inter-rater reliability was 96%. Owing to the small number of studies fulfilling the inclusion criteria, we decided to keep the study with high risk in the review.

### Data Extraction

Data extraction included (1) study design; (2) choice of the control condition; (3) sample characteristics in terms of sample size and justification of the size in power analysis, gender ratio, age, and mental health problems; (4) content of EMI in terms of specific CBT strategies; (5) EMI delivery characteristics in terms of mode, duration, and frequency; (6) feasibility in terms of acceptance, satisfaction, and helpfulness rates; (6) efficacy or effectiveness in terms of improvement in primary outcomes; and (7) outcome measures used in the studies.

### Analysis

Owing to the heterogeneity of the designs and objectives of the included studies, quantitative analysis was possible only in a few cases, for instance, for sample characteristics, delivery characteristics, and feasibility measures. However, in many aspects, such as effectiveness and effectiveness measures, quantitative analysis was not possible and, instead, a narrative approach for the summary of the qualitative findings was chosen.

## Results

### Included Studies

**Figure 1:**
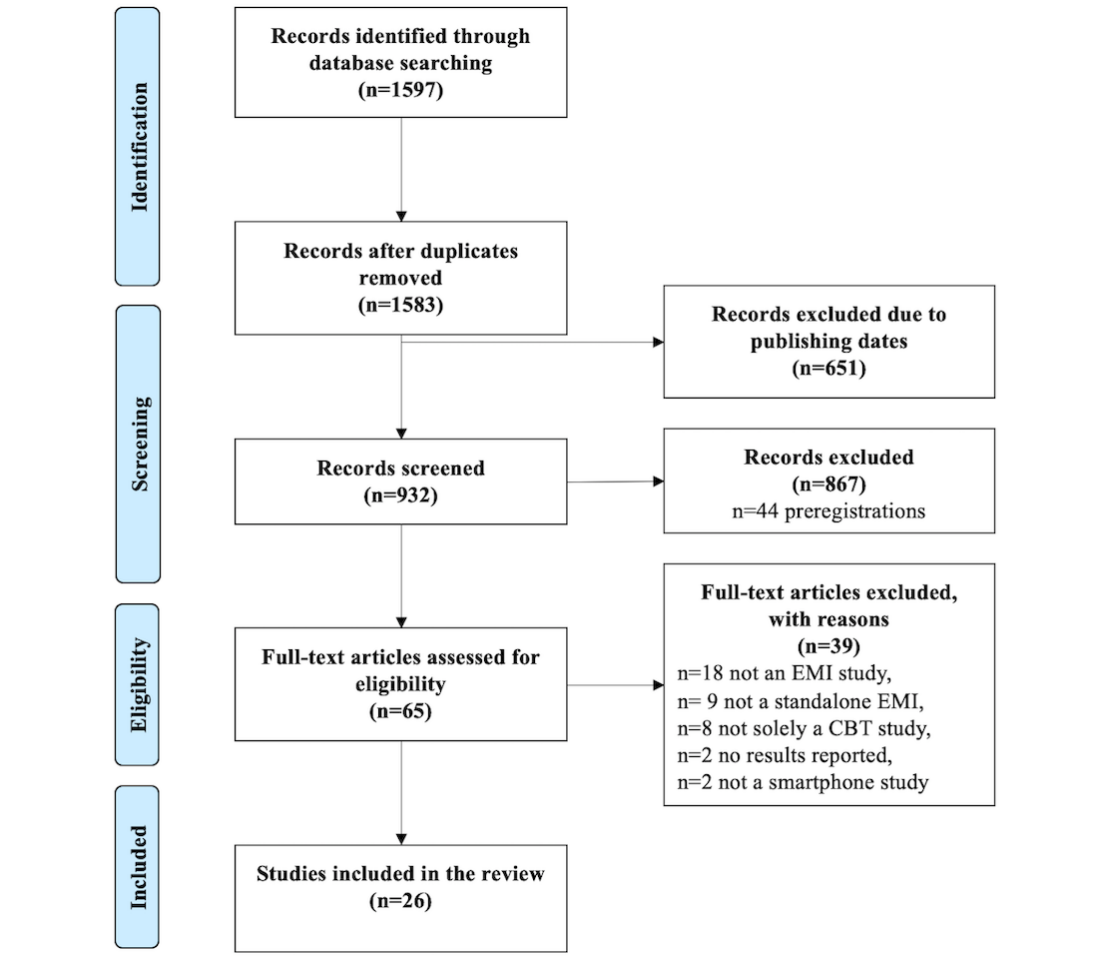
PRISMA (Preferred Reporting Items for Systematic Reviews and Meta-Analyses) flow diagram.

Inclusion criteria resulted in the identification of 26 articles (see Figure 1 for the complete literature flow chart). Of those, 3 [28-30] described the outcomes of 1 trial. For this particular trial, we only included data from the latest publication to avoid bias or duplication. Thus, our results are based on n=24 studies.

### Study Design and Control Conditions

A variety of study designs were employed to investigate the effects of EMIs: 2-, 3-, or 4-armed RCTs (12 studies [30-41]) as well as a nonrandomized trial with a control group (1 study [42]) for between-group comparisons or case studies and 1-group-only studies (11 studies [43-53]) for within-group or intraindividual comparisons (Table 1). Control conditions were selected based on the research questions of a given study. The control conditions in the studies reviewed here include (1) another app with different content (8 studies), (2) a waiting list (4 studies), (3) ecological momentary assessment (EMA)—participants were involved in self-monitoring only (2 studies), (4) encouragement to visit helpful websites or call hotlines (2 studies), (5) computer-based treatment (the same content of the intervention but delivered via internet browser; 1 study), (6) real-life training provided by a qualified person (eg, meditation training; 1 study), and (7) treatment as usual (1 study).

**Table 1:** Summary of the study design and control conditions.

Study	Names of apps	RCT^a^	Number of arms	Control condition	Type of control condition
Arevian et al, 2018 [43]	B-RESILIENT app	—^b^	—	—	—
Bakker et al, 2018 [32]	Mood Prism, Mood Mission, Mood Kit	✓^c^	4	✓	AA^d^, AA, WL^e^
Birney et al, 2016 [31]	Mood Hacker	✓	2	✓	E^f^
Christoforou et al, 2017 [33]	Agoraphobia Free, Stress Free	✓	2	✓	AA
Dahne et al, 2019 [34]	¡Aptívate!, iCouch CBT	✓	3	✓	AA, TAU^g^
Donker et al, 2019 [35]	ZeroPhobia	✓	2	✓	WL
Donovan et al, 2016 [44]	BodiMojo	—	—	—	—
Dulin et al, 2014 [45]	Buddy Steps	—	—	—	—
Hidalgo-Mazzei et al, 2018 [46]	OpenSIMPLe	—	—	—	—
Horsch et al, 2017 [36]	Sleepcare app	✓	2	✓	WL
Hur et al, 2018 [37]	Todac Todac app	✓	2	✓	EMA^h^
Levin et al, 2018 [38]	—	✓	3	✓	AA, WL
Morris et al, 2010 [47]	Mood Map	—	—	—	—
Morrison Wylde et al, 2017 [48]	Headspace	—	2	✓	RLT^i^
Prada et al, 2016 [49]	EMOTEO	—	—	—	—
Pratap et al, 2018 [30]	iPST, EVO, Health Tips	✓	3	✓	AA, AA
Roncero et al, 2018 [50]	GGRO	—	—	—	—
Roy et al, 2015 [39]	GETSmart	✓	2	✓	E
Schlosser et al, 2018 [42]	PRIME-D	—	3	—	AA, AA
Shrier et al, 2017 [52]	—	—	—	—	—
Stoll et al, 2017 [51]	REACH app	—	—	—	—
Versluis et al, 2018 [40]	—	✓	3	✓	WL, EMA
Watts et al, 2013 [41]	Get Happy	✓	2	✓	C^j^
Wen et al, 2017 [53]	Headspace	—	—	—	—

^a^RCT: randomized controlled trial.

^b^Not applicable or no information.

^c^Used in the study design.

^d^AA: another app with different content.

^e^WL: waiting list.

^f^E: encouragement to visit helpful websites or call hotline in case of a difficult situation.

^g^TAU: treatment as usual.

^h^EMA: app with ecological momentary assessment.

^i^RTL: real-life training with a qualified person.

^j^C: computer-based treatment.

### Target Populations and Mental Health Problems

EMIs were implemented in nonclinical populations, including healthy participants, and participants with subthreshold symptoms of a mental disorder (10 studies). In these studies, EMIs aimed to tackle a variety of problems, including high self-criticism or high levels of stress in the workplace (eg, of medical doctors, novice nurses, and corporation workers), all of which were assumed to increase the risk of developing a mental health disorder. In additional studies with healthy populations, EMIs addressed subthreshold posttraumatic stress disorder. EMIs were also implemented for the treatment of mental health issues in clinical populations (14 studies), including patients with depression, insomnia, bipolar disorder, schizophrenia, alcohol addiction, borderline personality, agoraphobia, or obsessive-compulsive disorder (Table 2).

**Table 2:** Sample characteristics.

Study	Population	Final sample size (n)	Power analysis	Age (years)	Age (years), mean (SD)	Gender (female %)
Arevian et al, 2018 [43]	Healthy	28	—^a^	18+	—	40 (11)
Bakker et al, 2018 [32]	Healthy and subthreshold depression and anxiety in screening	198	✓^b^	18-76	34.2 (14.2)	72.7 (144)
Birney et al, 2016 [31]	Clinical: depression	300	✓	18+	—	—
Christoforou et al, 2017 [33]	Clinical: agoraphobia	142	✓	18+	—	—
Dahne et al, 2019 [34]	Clinical: depression	42	—	18+	36.05 (11.44)	67 (28)
Donker et al, 2019 [35]	Clinical: acrophobia	193	✓	18-65	41.33 (13.68)	66.9 (129)
Donovan et al, 2016 [44]	Healthy	16	—	13-22	16.9 (1.3)	65 (10)
Dulin et al, 2014 [45]	Clinical: alcohol use disorder	28	—	22-45	33.6 (5.6)	46 (13)
Hidalgo-Mazzei et al, 2018 [46]	Clinical: bipolar disorder	103	—	18+	36.59 (11)	63.2 (65)
Horsch et al, 2017 [36]	Clinical: insomnia	151	✓	18-80	39.66 (13.44)	62.3 (94)
Hur et al, 2018 [37]	Clinical: depression	34	—	18-35	23.71 (3.26)	88 (30)
Levin et al, 2018 [38]	Healthy: high self-criticism	87	—	18-52	22.76 (7.02)	69 (60)
Morris et al, 2010 [47]	Healthy: high level of stress	8	—	30-48	37 (5.75)	—
Morrison Wylde et al, 2017 [48]	Healthy: medical workers	95	✓	23-50	—	92 (87)
Prada et al, 2016 [49]	Clinical: borderline	16	—	18-50	30.5 (93)	100 (16)
Pratap et al, 2018 [30]	Clinical: depression	345	—	18-70	34.9 (10.72)	77.1 (266)
Roncero et al, 2018 [50]	Clinical: obsessive-compulsive disorder	20	—	19-26	—	80 (16)
Roy et al, 2015 [39]	Subthreshold PTSD^c^	13	—	—	—	—
Schlosser et al, 2018 [42]	Clinical: depression	36	—	18+	31.33 (12.4)	78 (28)
Shrier et al, 2017 [52]	Clinical: depression	16	—	16-23	19.6 (—)	100 (16)
Stoll et al, 2017 [51]	Healthy	132	—	8-12	9.65 (0.82)	62.9 (83)
Versluis et al, 2018 [40]	Healthy: high level of stress	128	✓	18+	—	—
Watts et al, 2013 [41]	Clinical: depression	35	—	18-63	41 (12.38)	80 (28)
Wen et al, 2017 [53]	Healthy: medical workers	30	—	—	—	90 (27)

^a^No information.

^b^Power analysis was conducted.

^c^PTSD: posttraumatic stress disorder.

Sample sizes varied widely, from n=8 to n=348. Seven studies reported a power analysis to justify their sample size. The age range varied from 8 to 80 years. Over one-third of the studies did not report the age range of participants, informing only that they were aged 18 years or older. Five studies did not report the sex composition of their sample. Those that did included a majority (>70%) of female participants. This was due, in part, to the populations of interest. For instance, 1 study was conducted in novice nurses—a profession that is dominated by women. Two other studies included only women. In summary, the sample characteristics of current EMI studies are heterogeneous, and the quality of sample descriptions varies widely across studies.

### Content

Cognitive behavioral techniques provide a range of possible interventions that address diverse and complex patient needs [54]. CBT comprises techniques addressing psychological mechanisms that underpin negative and potentially harmful thoughts and beliefs (eg, reflection and cognitive restructuring) and actions (eg, behavioral activation or social skills training) endorsed by patients.

The most frequently used CBT strategies were cognitive restructuring, including reappraisal (14 studies), self-monitoring (13 studies), reflection (10 studies), and relaxation (8 studies). Cognitive restructuring identifies and changes the maladaptive thoughts and beliefs and reevaluates a given situation [15].

Self-monitoring is usually implemented in apps that contain an EMA module and is often described as a key component of successful therapy for many mental health conditions [55,56]. By getting access to their self-reported data, users become self-aware; manage their symptoms; better understand their mood, behavior, or illness; and work on these factors of mental well-being. Reflection is a metacognitive process that allows an individual to increase his or her psychological mindedness and another central process in CBT [57,58]. The reflection process requires the user to look at her or his experiences from a distance and often poses a starting point for many other therapeutic strategies (eg, reappraisal and gratitude). In addition, reflection was used, for instance, as an outcome of self-monitoring [37,44].

The use of digital technologies opens a new door to deliver CBT strategies. Apart from basic solutions, such as sending messages [52] or text-based scenarios [37,50], novel approaches were employed in the reviewed studies. For instance, reflection and cognitive restructuring were presented in a fabular comic story of the main character who had depression [41]. A similar solution was employed in the Agoraphobia free app, which was game based and presented a virtual character who needed to meet the virtual therapist to work on reflection and cognitive restructuring [33]. Relaxation and meditation exercises were delivered via audio and video tools [49,53]. Self-monitoring outcomes were presented, for instance, as *mood cloud*, providing a visual representation of the participant's self-reported mood [44], or in a calendar view, to allow the user to track their behavior day-by-day or even hour-by-hour [36]. One novel solution combines a mobile app with virtual reality (VR) to treat acrophobia with exposure [35] or a platform allowing users to contact each other to provide social support that enhances behavioral activation [42]. All apps in the studies reviewed here used more than one strategy to improve mental health (Table 3).

**Table 3:** Cognitive behavioral therapy techniques implemented in ecological momentary interventions.

Study	BA^a^	CR^b^	DF^c^	DSC^d^	DST^e^	EXP^f^	MDF^g^	PS^h^	PE^i^	RF^j^	RLX^k^	SMN^l^	SST^m^
Arevian et al, 2018 [43]	—^n^	✓^o^	—	—	—	—	—	—	—	—	—	—	✓
Bakker et al, 2018 [32]^p^	✓	—	—	—	—	—	—	—	✓	—	—	✓	—
Birney et al, 2016 [31]	✓	✓	—	—	—	—	✓	—	—	—	—	✓	—
Christoforou et al, 2017 [33]	—	✓	—	—	✓	✓	—	—	✓	✓	✓	—	—
Dahne et al, 2019 [34]	✓	—	—	—	—	—	—	—	✓	✓	—	✓	—
Donker et al, 2019 [35]^p^	—	—	—	—	—	✓	—	—	✓	—	—	—	—
Donovan et al, 2016 [44]	—	✓	—	—	—	—	✓	—	—	✓	—	✓	—
Dulin et al, 2014 [45]	✓	—	—	—	—	—	—	✓	✓	—	—	✓	✓
Hidalgo-Mazzei et al, 2018 [46]	—	—	—	—	—	—	—	—	✓	—	—	✓	—
Horsch et al, 2017 [36]	✓	—	—	—	—	—	—	—	✓	—	✓	✓	—
Hur et al, 2018 [37]	—	✓	—	✓	—	—	—	—	—	✓	—	✓	—
Levin et al, 2018 [38]	—	✓	✓	—	—	—	—	—	—	✓	—	—	—
Morris et al, 2010 [47]	—	✓	—	—	—	—	—	—	—	—	✓	✓	—
Morrison Wylde et al, 2017 [48]	—	—	—	—	—	—	✓	—	—	✓	✓	—	—
Prada et al, 2016 [49]	—	—	—	—	✓	—	✓	—	—	—	✓	✓	—
Pratap et al, 2018 [30]	—	✓	—	—	—	—	—	✓	✓	—	—	—	—
Roncero et al, 2018 [50]	✓	✓	—	—	—	—	—	—	—	—	—	—	—
Roy et al, 2015 [39]	—	✓	—	—	—	—	—	—	✓	✓	✓	—	✓
Schlosser et al, 2018 [42]	✓	—	—	—	—	—	✓	—	✓	—	—	—	✓
Shrier et al, 2017 [52]	—	✓	—	—	—	—	—	—	—	✓	—	✓	—
Stoll et al, 2017 [51]	—	✓	—	—	—	—	—	—	—	—	✓	✓	—
Versluis et al, 2018 [40]	—	✓	—	—	—	—	✓	—	—	—	—	✓	—
Watts et al, 2013 [41]	—	✓	—	—	—	—	—	—	✓	✓	—	—	✓
Wen et al, 2017 [53]	—	—	—	—	—	—	✓	—	—	✓	✓	—	—

^a^BA: behavioral activation.

^b^CR: cognitive restructuring, including reappraisal.

^c^DF: defusion.

^d^DSC: distancing.

^e^DST: distraction.

^f^EXP: exposure.

^g^MDF: mindfulness.

^h^PS: problem solving.

^i^PE: psychoeducation.

^j^RF: reflection.

^k^RLX: relaxation.

^l^SMN: self-monitoring.

^m^SST: social skills training.

^n^Technique not implemented in the ecological momentary intervention.

^o^Technique implemented in the ecological momentary intervention.

^p^Insufficient information was provided—probably more modules were implemented.

### Delivery

The most common way to deliver EMIs on a smartphone is a mobile app, but ever-growing possibilities are emerging with technological advances. For example, mobile apps can be combined with wristbands to track indicators of physiological function (eg, heartbeat), GPS to track geolocation, or VR.

The duration of interventions ranged from 2 weeks to 6 months, with a median of 30 days. A frequent option was to send prompts to remind participants either about using EMI or about completing the EMA (17 studies). The number of prompts with EMA or reminders varied from 5 per day to once every week (Table 4). In 4 apps, prompts were sent only when the participant forgot to use the app for a longer, predefined period. Another way to deliver EMIs was to allow participants to use them anytime they needed to or once per day.

**Table 4:** Characteristics of ecological momentary intervention delivery.

Study	Intervention duration in days	Prompts sent	Number of prompts per day	Trigger
Arevian et al, 2018 [43]	28	✓^a^	1	Time based
Bakker et al, 2018 [32]	30	✓ 1/3^b^	N/I^c^	N/A^d^
Birney et al, 2016 [31]	42	✓	1	N/I
Christoforou et al, 2017 [33]	84	✓	0.14	N/I
Dahne et al, 2019 [34]	56	✓	0.14	Participant
Donker et al, 2019 [35]	21	✓	0.14	Participant
Donovan et al, 2016 [44]	30	N/A	N/A	Participant
Dulin et al, 2014 [45]	42	✓	1	Random
Hidalgo-Mazzei et al, 2018 [46]	168	✓	1	EMA^e^
Horsch et al, 2017 [36]	84	✓	1 AL^f^	Depending on data^g^
Hur et al, 2018 [37]	21	✓	0.5 ANU^h^	EMA
Levin et al, 2018 [38]	14	✓	4	Participant and random
Morris et al, 2010 [47]	30	✓	N/I	Participant
Morrison Wylde et al, 2017 [48]	28	N/A	N/A	N/A
Prada et al, 2016 [49]	168	N/A	N/A	N/A
Pratap et al, 2018 [30]	84	✓	1	Participant
Roncero et al, 2018 [50]	15	✓	1	N/I
Roy et al, 2015 [39]	42	✓	1	Depending on data
Schlosser et al, 2018 [42]	56	N/A	N/A	Participant
Shrier et al, 2017 [52]	28	✓	5	Random
Stoll et al, 2017 [51]	1	N/A	N/A	N/A
Versluis et al, 2018 [40]	26	✓	5	Random
Watts et al, 2013 [41]	56	N/A	N/A	N/A
Wen et al, 2017 [53]	30	N/A	N/A	Participant

^a^Prompts were sent by ecological momentary intervention.

^b^1/3: in 1 of the 3 apps introduced in the study (when not marked, all apps introduced in the study were sending beeps).

^c^N/I: no information.

^d^N/A: not applicable.

^e^EMA: ecological momentary assessment.

^f^AL: at least (depending on participants’ reports).

^g^Depending on data: depending on participants’ report other than ecological momentary assessment (eg, sleep hygiene).

^h^ANU: beeps were sent only when app was not used.

Triggers can be divided into 3 categories: time based, event based, and randomized within a specific time frame. In 10 studies, event-based triggers were employed to allow participants to trigger EMI themselves. For example, participants were instructed to use the app anytime they felt they might benefit from the EMI (Table 4). Another trigger from this category was EMA (used in 2 studies): when problems or symptoms were reported (eg, low mood or above-threshold stress level), the participant received an EMI. This just-in-time adaptive intervention (JITAI) aims to provide the right type or amount of support, at the right time, by adapting to an individual's changing internal and contextual state [59]. Four studies used randomized prompt-sending as an EMI trigger to avoid prepared answers from participants. One app sent prompts every morning.

The EMI frequency varied among studies. The range of the frequency of EMI delivery was from 3 times per day to once per week. Four studies did not report the number of delivered EMIs.

Taken together, the frequency, triggers, and duration of the intervention varied and depended on the target group, targeted problem, and CBT techniques implemented. A few studies did not report this information, including default or the average number of prompts per participant or duration of intervention for different participants.

### Feasibility

For this review, feasibility was indexed by compliance rate and participants’ satisfaction with the intervention.

Compliance reporting was variable. First, the definition of compliance differed across studies. Some researchers reported how many training sessions participants completed, how many prompts participants answered, or the number of participants who did not drop out of the study. Others set thresholds, such as reacting to at least one or two prompts and subsequently excluded all participants with lower adherence. For the purpose of this review, we defined *compliance rate* as the number of reactions to prompts. Nineteen studies did not report compliance. None of the studies with healthy samples reported compliance rates.

In other studies, compliance ranged from 33.8% to 93.3% (7 studies; mean 64, SD 22). There was no relationship between compliance rate and duration as well as the overall time cost of the intervention. For instance, one study reported a compliance rate of 55.6% for a 15-day intervention with 1 prompt per day [47], and another reported a compliance rate of 33.8% for a 168-day intervention with 1 prompt per day [43], whereas other studies reported a compliance rate of 90.5% for a 56-day intervention with 1 prompt per week [31] or a compliance rate of 93.3% for a 42-day intervention with 1 prompt per day [28]. All studies with higher than average compliance rates included participants with depression. Lower compliance rates were reported in a study of participants with bipolar disorder.

Satisfaction and helpfulness rates were underreported (satisfaction was reported in 7 studies and helpfulness in 4 studies) but rated positively (mean perceived satisfaction 72.6%, SD 17.2 and mean perceived helpfulness 70.8%, SD 15.3). Helpfulness was rated highest by individuals with borderline personality disorder and healthy samples with high self-criticism. Healthy populations, especially adolescents, were generally more satisfied with the EMIs compared with clinical populations (Table 5). The least satisfied were users with bipolar disorder; however, only 1 study was conducted on such a sample. Discrepancies were found in satisfaction rates reported by depressed samples, ranging from 46% and 54% (which were the lowest reported rates) to 91.8% (which was one of the highest rates). However, only 3 of 7 studies that delivered EMI to depressed populations reported these numbers.

**Table 5:** Feasibility, efficacy, and effectiveness.

Study	Compliance in %	Helpfulness in %	Satisfaction in %	Primary outcome measure	Improvement in primary outcome	Secondary outcome measure
Arevian et al, 2018 [43]	—^a^	—	—	—	—	—
Bakker et al, 2018 [32]	—	—	—	PHQ^b^, GAD^c^, WEMWBS^d^	✓^e^	ESAS-R^f^, CSES^g^, MHLQ^h^
Birney et al, 2016 [31]	93	—	46	PHQ	—	BADS^i^, ATQ-R^j^, KT^k^
Christoforou et al, 2017 [33]	52	—	—	PAS^l^	✓	F^m^
Dahne et al, 2019 [34]	91	—	—	PHQ	✓	F
Donker et al, 2019 [35]	—	—	—	AQ^n^	✓	ATHQ^o^, BDI-II^p^, IPQ^q^, PHQ, M^r^, SUS^s^
Donovan et al, 2016 [44]	—	64	92	—	—	—
Dulin et al, 2014 [45]	—	—	—	TLFB^t^	✓	F
Hidalgo-Mazzei et al, 2018 [46]^u^	33.8	—	62	WHO-5^v^, SF-36^w^	✓	F
Horsch et al, 2017 [36]	49.7	—	—	ISI^x^	✓	PSQI^y^, DBAS-16^z^, HADS^aa^, CES-D^ab^
Hur et al, 2018 [37]	—	—	—	DAS^ac^, BDI-II, STAI^ad^, RSES^ae^	✓	QoL^af^
Levin et al, 2018 [38]	—	78 and 83	81 and 81	FSCRS^ag^	✓	DASS^ah^, WSAS^ai^
Morris et al, 2010 [47]	—	—	—	I^aj^, EMASS^ak^	✓	—
Morrison Wylde et al, 2017 [48]	—	—	—	CFST^al^	✓	LEC^am^, PCL-C^an^, FFMQ^ao^
Prada et al, 2016 [49]	—	82	—	EMAAT^ap^	✓	—
Pratap et al, 2018 [30]^u^	—	—	—	PHQ, SDS^aq^	—	CR^ar^
Roncero et al, 2018 [50]	56	—	—	OCI-R^as^, OBQ^at^	✓	ROCI^au^, PROCSI^av^, DASS
Roy et al, 2015 [39]	—	—	—	PCL^aw^	✓	PHQ, GAD
Schlosser et al, 2018 [42]	—	—	72	PHQ, SDS	✓	—
Shrier et al, 2017 [52]	—	—	—	—	—	—
Stoll et al, 2017 [51]	—	—	92	—	—	—
Versluis et al, 2018 [40]	—	47	—	HR^ax^	—	IPANAT^ay^, IAT^az^, ERI^ba^, PSWQ^bb^, GAD, PHQ, FFMQ, CEQ^bc^, EMASE^bd^, F
Watts et al, 2013 [41]	69	—	54	PHQ	✓	K-10^be^, BDI-II, CEQ, SDS, ERS^bf^, HRS^bg^
Wen et al, 2017 [53]	—	—	—	PANAS^bh^, FMI^bi^	✓	—

^a^No information.

^b^PHQ: Patient Health Questionnaire.

^c^GAD: Generalized Anxiety Disorder Scale.

^d^WEMWBS: Warwick-Edinburgh Mental Well-Being Scale.

^e^Study with improvement in the primary outcomes. Improvement in primary outcomes=results statistically significant in at least one measure of the primary outcomes.

^f^ESAS-R: Emotional Self-Awareness Scale-Revised.

^g^CSES: Coping Self-Efficacy Scale.

^h^MHLQ: Mental Health Literacy Questionnaire.

^i^BADS: Behavioral Activation for Depression Scale.

^j^ATQ-R: Automatic Thoughts Questionnaire-Revised.

^k^KT: knowledge test.

^l^PAS: Panic and Agoraphobia Scale.

^m^F: feasibility measures.

^n^AQ: Acrophobia Questionnaire.

^o^ATHQ: Attitudes Toward Heights Questionnaire.

^p^BDI-II: Beck Depression Inventory II.

^q^IPQ: Igroup Presence Questionnaire.

^r^M: mastery.

^s^SUS: System Usability Scale.

^t^TLFB: The Timeline Followback.

^u^To provide information about efficacy, we reversed the order of the outcomes.

^v^WHO-5: World Health Organization 5-point Well-Being Index.

^w^SF-36: Short Form Health Survey.

^x^ISI: Insomnia Severity Index.

^y^PSQI: Pittsburgh Sleep Quality Index.

^z^DBAS-16: Dysfunctional Beliefs and Attitudes about Sleep.

^aa^HADS: Hospital Anxiety and Depression Scale.

^ab^CES-D: Center of Epidemiologic Studies Depression Scale.

^ac^DAS: Dysfunctional Attitude Scale.

^ad^STAI: State-Trait Anxiety Inventory.

^ae^RSES: Rosenberg Self-Esteem Scale.

^af^QoL: quality of life.

^ag^FSCRS: Forms of Self-Criticism and Self-Reassurance Scale.

^ah^DASS: Depression, Anxiety, and Stress Scale.

^ai^WSAS: Work and Social Adjustment Scale.

^aj^I: interview.

^ak^EMASS: Ecological Momentary Assessment-Stress Scale.

^al^CFST: Compassion Fatigue Self-Test.

^am^LEC: Life Events Checklist.

^an^PCL-C: Posttraumatic Stress Disorder Checklist-Civilian Version.

^ao^FFMQ: Five Facet Mindfulness Questionnaire.

^ap^EMAAT: Ecological Momentary Assessment-Aversive Tension Scale.

^aq^SDS: Sheenan Disability Scale.

^ar^CR: comparison of recruitment and engagement in a fully remote trial of individuals with depression who either self-identify as Hispanic/Latino or not.

^as^OCI-R: Obsessive-Compulsive Inventory-Revised.

^at^OBQ: Obsessive Beliefs Questionnaire.

^au^ROCI: Relationship Obsessive-Compulsive Inventory.

^av^PROCSI: Partner-Related Obsessive-Compulsive Symptoms Inventory.

^aw^PCL: Posttraumatic Stress Disorder Checklist.

^ax^HR: heart rate.

^ay^IPANAT: Implicit Positive and Negative Affect Test.

^az^IAT: Implicit Association Test.

^ba^ERI: Effort-Reward Imbalance Questionnaire.

^bb^PSWQ: Penn State Worry Questionnaire.

^bc^CEQ: Credibility/Expectancy Questionnaire.

^bd^EMASE: Ecological Momentary Assessment-Stress and Explicit Affect.

^be^K-10: Kessler 10-Item Psychological Distress Scale.

^bf^ERS: Environment Rating Scale.

^bg^HRS: Homework Rating Scale.

^bh^PANAS: Positive and Negative Affect Scale.

^bi^FMI: Freiburg Mindfulness Scale.

### Efficacy and Effectiveness

Efficacy or effectiveness was reported in 21 studies. In the 8 studies reviewed here, the authors used both these terms alternately to report the outcomes of EMI. Hence, both effectiveness and efficacy are reported in this review, and the particular term is used as indicated by the authors of the studies.

In total, 16 of the 21 interventions included in our review reported evidence of a significant reduction in mental disorder symptoms (Table 5).

Nine studies reported effect sizes (Cohen *d*). In the EMI study for insomnia, the authors found significant interaction effects between time and condition on the primary outcome measures (*d*=−0.66) and sleep efficiency (*d*=0.71) [33]. A significant effect on insomnia severity was also recorded at the 3-month follow-up. In the study investigating high self-criticism, effect sizes for between-group comparison and for all primary outcome measures ranged from 0.46 to 0.72 (except inadequate self-criticism, which was nonsignificant) [35]. Another study assessing the effectiveness of EMI in individuals with acrophobia found a between-group effect size *d*=1.14 [32].

The within-group effect sizes varied from *d*=0.47 to *d*=0.64 and *d*=0.67 for depressed samples and changes in overall health, as indexed by the Patient Health Questionnaire. One study found effect sizes of *d*=0.77 for measures of mindfulness in a medical workers’ sample and a moderate effect size *d*=0.38 for positive affect [50]. A large effect (*d*=1.0) was found in the study on a population with alcohol use disorder [42]. The only effect sizes that were small or null were found in the study investigating EMI helping in obsessive-compulsive disorder [47]. Taken together, the effect sizes for a reduction in primary outcomes mostly varied from moderate to large, with large effects found for the majority of the reviewed EMIs.

The most common method to assess efficacy and effectiveness was with questionnaires, and almost all the studies using this method found statistically significant differences (in both between- and within-group comparisons) in the primary outcomes (Table 5). With respect to other measures of primary outcomes, 1 study used heart rate [37] and 2 other studies calculated effectiveness based on EMA data [44,46]. Although the outcomes based on EMA were statistically significant, there were no effects found based on physiological readouts.

As for secondary outcomes, 1 of the studies employed a knowledge test about depression and found statistically significant positive effects for the program on information and knowledge [28]. In another study, the authors added an Implicit Positive and Negative Affect Test to assess the level of unconscious stress and found that implicit stress did decrease over time for all participants, independent of the intervention [37].

## Discussion

### Principal Findings

We reviewed 26 studies that investigated the feasibility, efficacy, and effectiveness of standalone CBT-based EMIs to increase mental health. Results show that EMIs can be successfully delivered via smartphones, significantly increase well-being among users, and reduce symptoms of mental disorders. Designs and quality of the studies reviewed using a rational app design were heterogeneous. Across studies, EMIs were generally accepted by users with various age, sex, education background, and professions and were shown to be effective treatments for a broad range of psychological symptoms.

CBT assumptions constitute an evidence-based basis for EMIs, especially with the use of techniques involving self-monitoring, reflection, cognitive restructuring, and relaxation. Such techniques were mostly employed in combination, and users were sometimes provided with a personal choice of which approach they preferred.

Many studies investigated the efficacy of reducing symptoms of depression in clinical populations. The Todac Todac app, for instance, significantly reduced both depression and anxiety symptoms [37]. The Get Happy program was associated with stable reduction of depressive symptoms, including a demonstration of stability at 3 months follow-up [41]. Promising results were also shown in studies with healthy samples, where an increase in positive well-being and higher emotional self-awareness along with self-efficacy as a result of using EMI were reported. Here, the MoodKit, MoodMission, and MoodPrism apps were effective in decreasing mild depressive and anxiety symptoms [32]. Headspace is a mindfulness app that was used in 2 studies with medical workers and tested against a control group that received face-to-face mindfulness classes. Users engaging in Headspace reported higher self-awareness in their actions, less burnout, and more compassion satisfaction compared with the group receiving face-to-face mindfulness training [47]. In another study of the Headspace app, a single group design was chosen. This study reported heightened mindfulness skills and more positive emotions as a result of using Headspace post-EMI versus pre-EMI in medical workers [53].

### Clinical Perspective

From a practical clinical perspective, EMIs may postpone the necessity of professional mental health treatment by a psychiatrist or psychologist, eg, for individuals who struggle financially, have limited access to such services in their place of living, or due to health reasons cannot attend psychotherapy [32,51]. On the basis of this review and previous studies, EMIs may be specifically helpful for individuals who are facing temporary problems, for instance, in the context of personal or professional change or other transitioning periods. Moreover, symptoms of mental health are still affected by considerable stigmatization [60], and EMIs may help reduce the fear of stigma as psychological support can be obtained without seeking treatment in a hospital setting. EMIs are also often a first step for many individuals to test personal preferences and affinity to psychological treatment and may lead to face-to-face or other professional therapeutic services later [61]. The worldwide long-lasting crisis of mental health care, manifested by poor accessibility of cost-free, state-funded psychotherapy, suggests another use of EMIs. Patients could use EMIs as a source of support while awaiting face-to-face treatment [62-64].

### EMIs in Context of Pandemics

In the context of COVID-19, the need to implement population-based behavioral measures to counter the rapid spread of the virus has led to home confinement; loss of individual behavioral routines, including exercise; and lack of social contact. Psychological needs are often underaddressed in such times of crises [8,65]. EMIs could be an ideally suited approach to reach a large number of individuals affected by mental health problems and to provide scalable global mental health solutions. It is also estimated, based on experiences with previous pandemics, that after the COVID-19 pandemic is over, such solutions will be needed to help populations with continued psychological symptoms, such as depression and anxiety [66]. Some populations might be at a specific risk of stress in this challenging time, especially doctors, nurses, or emergency personnel [67-69], and 2 of the trials reviewed here investigated effects on the medical staff. The high efficacy of the Headspace app tested in these populations of first responders and frontline workers is encouraging and indicates that mindfulness training delivered via a mobile app can significantly decrease work-related stress. EMIs could thus help to lower the risk of developing unfavorable mental health outcomes in such populations, which have recently been documented [70].

### Methodological Limitations

There are several methodological caveats in the studies reviewed here. The choice of control condition varied. Suggestions for the control groups to call hotlines or visit recommended websites imply heterogeneous and less tangible control groups than control groups that could use comparison apps. At the same time, comparing results from 2 different apps can be difficult if they target different symptoms or use different techniques. Little attention has been paid to the psychological mechanisms (eg, cognitive mediators) that allowed for this change in the outcome to develop. To increase the conclusiveness and reliability of studies assessing efficacy, effectiveness, and feasibility of EMIs, more RCTs with adequate control groups are needed [71]. Ideally, RCTs should be preceded by optimization studies, based, for instance, on MOST (Multiphase Optimization Strategy) [72] and SMART (The Sequential Multiple Assignment Randomized Trial) [73], which allow for choosing the right dosage and delivery of intervention based on predefined criteria, for example, individual characteristics of the user, characteristics of psychiatric population, and effectiveness of the intervention. Another valid concern is the reporting of EMI studies. For example, the majority (73%) of the studies did not report a formal power analysis. Consequently, studies might be underpowered, and their quantitative results have not been reported. However, some studies investigated the feasibility rather than efficacy and focused on issues of implementation, launch, and users’ experience with the app; thus, they might have been less concerned about power and sample size. Moreover, it must be noted that there might also be a bias in overestimating the effects of EMI. First, control conditions, if used, cannot simply be blinded, similar to psychotherapy studies. Second, the fact that almost all studies showed a positive effect on the primary outcome makes it likely that there is a publication bias toward positive results.

Despite these limitations, we indicated key strengths and found a number of standalone smartphone-based EMIs that were shown to be effective. It is also in line with findings from previous reviews in this field, although their focus differed, for instance, by focusing on blended therapies [24,25] or specific mental health problems [12,19-23].

### Recommendations for Future Development

Exciting future developments lie ahead in the field of EMIs. This is documented by >40 (pre)registered protocols, which we excluded (Figure 1) and which are likely to produce further evidence about the effectiveness of CBT-based standalone EMIs. Future developments should exploit the potential to scale these interventions and test their effects in larger and global populations in times of crises, such as the COVID-19 pandemic. Moreover, it is possible to combine EMIs with wearable technologies [74] to gather physiological data and other information for researchers and clinicians alike. EMIs can be used along passive sensing tracking patterns of smartphone usage, and by using context-aware assessment strategies that link the assessment of experiences to specific sensing events (like, for instance, physical distance to the others), would allow for the design of specific interventions in response to COVID-19 pandemic–induced mental health problems, especially induced by isolation whereas, at the same time, complying with the demanded pandemic containment policies. Here, mobile crowdsensing in combination with EMI would constitute the key technology to address this major mental health challenge. Ideally, these tools can be combined with machine learning to derive algorithms for accurate and precise prediction and selection of ecologically valid treatment options [75]. Machine learning, or more generally artificial intelligence techniques, could be useful in at least three ways. First, such approaches could identify individuals and/or time points where the intervention might be particularly important to avoid substantial degradation of mental health.

Second, such approaches could identify factors that critically affect effectiveness, which could subsequently help in personalizing EMIs both delivery mode and contentwise, and in enhancing decision making at precise time points while using personally tailored JITAIs.

Third, they could significantly decrease the time invested by health professionals and scientists who administer EMIs, such as in the study where clinicians manually adapted the number of EMIs delivered based on app usage [42].

### Conclusions

Taken together, our review showed that standalone CBT-based EMIs can be efficacious in reducing symptoms of psychological disorders in various situations. EMIs may support individuals outside the therapists’ room and could also be helpful when time and resource investment from mental health professionals are limited. At the same time, however, professional clinician-scientists’ input is invaluable in designing and implementing such EMIs. All EMIs reviewed here were based on a rational app design that translates key concepts and findings from CBT to these new interventions. They can provide scalable and evidence-based mental health support for large populations and be readily distributed.

## Appendix

Multimedia Appendix 1 Complete search strings.

## Abbreviations

CBT: cognitive behavioral therapy
EMA: ecological momentary assessment
EMI: ecological momentary intervention
JITAI: just-in-time adaptive intervention
mHealth: mobile health
RCT: randomized controlled trial
VR: virtual reality
WHO: World Health Organization
