# Supplementary material for: Standalone Smartphone Cognitive Behavioral Therapy–Based Ecological Momentary Interventions to Increase Mental Health: Narrative Review
Source: JMIR Mhealth Uhealth. 2020 Nov 12;8(11):e19836. doi: 10.2196/19836 (PMC7691088; doi:10.2196/19836)
Supplement: Multimedia Appendix 1 [file mhealth_v8i11e19836_app1.doc]

**Appendix 1. Complete search strings**

*“Ecological Momentary Intervention” search:* ('ecological momentary intervention' OR EMI OR 'just in time adaptive intervention' OR jitai OR 'digital intervention' OR 'educational app' OR 'in the moment assessment' OR 'technology-supported mini-intervention')

AND

*“Smartphone” search:* (smartphone OR mHealth OR 'mobile health' OR app OR SMS OR 'smartphone application' OR 'mobile app' OR 'digital intervention' OR 'mobile technology' OR 'personal health technology' OR telehealth)

AND

*“Cognitive behavioural therapy” search:* (CBT OR 'cognitive behavioural therapy' OR 'cognitive training' OR 'behavioural therapy' OR 'problem solving' OR 'behavioural activation' OR 'reappraisal' OR 'social skills training' OR psychoeducation OR 'self-monitoring' OR 'cognitive restructuring' OR exposure OR 'assertion training' OR mindfulness OR journaling OR relaxation OR 'successive approximation')

AND

*“Mental health” search:* (mental OR 'mental health' OR psychological OR psychology OR 'psychological treatment'
